# Multi-Stage Hough Space Calculation for Lane Markings Detection via IMU and Vision Fusion

**DOI:** 10.3390/s19102305

**Published:** 2019-05-19

**Authors:** Yi Sun, Jian Li, Zhenping Sun

**Affiliations:** The College of Intelligence Science and Technology, National University of Defense Technology, Changsha 410073, China; 15575191281@163.com (Y.S.); 13974913933@139.com (Z.S.)

**Keywords:** IMU, vision, classification networks, Hough transform, lane markings detection

## Abstract

It is challenging to achieve robust lane detection based on a single frame, particularly when complicated driving scenarios are present. A novel approach based on multiple frames is proposed in this paper by taking advantage of the fusion of vision and Inertial Measurement Units (IMU). Hough space is employed as a storage medium where lane markings can be stored and visited conveniently. The detection of lane markings is achieved by the following steps. Firstly, primary line segments are extracted from a basic Hough space, which is calculated by Hough Transform. Secondly, a CNN-based classifier is introduced to measure the confidence probability of each line segment, and transforms the basic Hough space into a probabilistic Hough space. In the third step, pose information provided by the IMU is applied to align previous probabilistic Hough spaces to the current one and a filtered probabilistic Hough space is acquired by smoothing the primary probabilistic Hough space across frames. Finally, valid line segments with probability higher than 0.7 are extracted from the filtered probabilistic Hough space. The proposed approach is applied experimentally, and the results demonstrate a satisfying performance compared to various existing methods.

## 1. Introduction

With the development of artificial intelligence, intelligent driving technology has made great progress thanks to the advancement of different kinds of sensors and powerful processors. It is a trend where intelligent vehicles play important roles in a safe and efficient transportation environment. Lane detection is an essential research field of intelligent driving, which could be employed to provide lane departure warning in Advanced Driver Assistance System (ADAS) and provide local road navigation for autonomous vehicles, especially when the GPS signal is disturbed.

Many methods are proposed to improve the performance of the lane-marking detection system. Line-segment extraction is a common step to detect lane markings. Well-known methods such as Hough Transform and LSD are very often employed. However, false positive results are given, and a post process is necessary to distinguish whether these line segments belong to lane markings or not. Geometry constraints (e.g., width-based constraints) are always used in this type of classification, but it is difficult to deal with particular kinds of line segments, such as those extracted from fences. Meanwhile, numerous end-to-end networks are proposed to detect lanes in images. Nevertheless, it is of difficulty to merge human logistical knowledge into the networks, and large amounts of labeled images are required.

Due to the disturbance of different kinds of noise, the detection results extracted from a single frame are not reliable for system control. Hence, the integration of sequential information is vital for the development of a robust method. On the other hand, though lane-marking tracking based on sequential information is already frequently employed, the movement information of the vehicle is usually obtained by estimation. As a result, the estimation error will reduce the tracking performance and make it hard to track lane markings at a large time scale. Therefore, obtaining more accurate vehicle information via Inertial Measurement Unit (IMU) is of great necessity.

To solve the problems mentioned above, a novel approach is proposed to extract lane markings by the fusion of vision and IMU. This work aims at obtaining a reliable Hough space which measures each line segment with a probability value. Finally, line segments with high probability values will be extracted from this Hough space. We divide this approach into two steps as follows:

**Constructing primary probabilistic Hough space:** a primary probabilistic Hough space is extracted from a single frame, which measures each line segment with a probability value. In this section, an efficient Hough Transform with edge gradient constraints [1] is employed for line-segment extraction and a CNN-based classifier is proposed for line-segment classification. The proposed probabilistic Hough space is constructed by the outputs of this classification network and each point in this probabilistic space describes the confidence possibility of the corresponding line segment. A threshold ξ (which is set to 0.7) is used to choose the valid line segments from the probabilistic Hough space. It is necessary to mention that, because Hough space makes it convenient for storing the results across frames, we construct a primary probabilistic Hough space to record the classification results of each frame.

**Filtering probabilistic Hough space across frames by IMU and vision data fusion:** due to the disturbance of occlusion, vehicle movement, and classification error, the primary probabilistic Hough space extracted from a single frame is not reliable. For example, the change of vehicle pose significantly could affect the classification results of the corresponding line segments. Consequently, the same lane markings might have different values in the probabilistic Hough space. To solve this, sequential information is included, and a Kalman Filter is employed to smooth the probabilistic Hough space across frames. While the vehicle is moving, the line segments extracted from images always have different positions in Hough space at different times, though they lie on the same lane markings. Movement information provided by the IMU makes it possible to align previous and current line segments in the current Hough space, which is essential for the filtering process. The final filtered probabilistic Hough space is used to extract the final line segments. Line segments with low probability value will be eliminated and those with high value will be kept and tracked.

**Figure 1:**
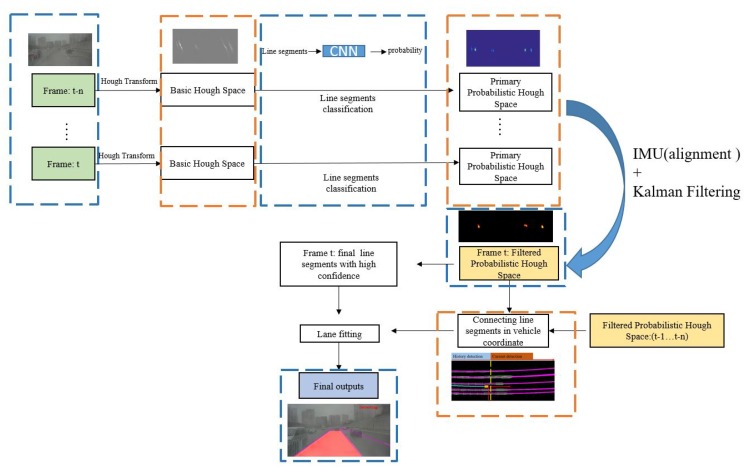
Workflow of the proposed approach: Hough Transform and Classification networks are used to extract the primary probabilistic Hough space. Kalman filter is introduced to smooth the probabilistic Hough space across frames, where sequential information is employed. Movement information provided by IMU is applied to make the previous line segments aligned in the same Hough space. The final filtered probabilistic Hough space is used to extract the final line segments with high probability value. By connecting valid line segments in the vehicle coordinates which are detected at different times, lane fitting could be solved with more sequential information and the final result would be more robust.

This paper consists of 6 sections. Related works will be introduced in Section 2. Section 3 describes the construction of the primary probabilistic Hough space depending on single frame. In Section 4, the primary probabilistic Hough space is filtered across frames by the fusion of IMU and vision data. The discussion of detailed experiments and conclusions are presented in Section 5 and Section 6, respectively. Figure 1 shows the workflow of the proposed method.

## 2. Related Works

Lane detection plays a fundamental role in current intelligent driving systems such as ADAS or autonomous driver systems. A large amount of vision-based methods has been proposed.

### 2.1. Conventional Algorithms without CNNs

In conventional lane-detection approaches, edge is a common and important feature for the extraction of lane markings. In [2,3,4], Canny is used to extract and locate the edge position in image. Many pre-processing algorithms are proposed to strengthen the feature of lane markings. In [2], an LDA model is applied to make it more distinguishable between the lane markings and background in RGB color space. A brightness stretching function named PLSF is proposed in [3] which makes lane markings become clearer than before. Each edge extraction method has its own strength and weakness, so [5] combines different strategies and uses local thresholds to extract edges, which make the edge extraction more robust. Prior information and top-to-bottom constraints are actually useful for eliminating false detection. For example, meaningful edge points are always located in the neighbor of line segments. Thus, in [4], a two-stage feature extraction method is proposed.

Hough Transform is a classical and robust approach to extract line segments from image. To purify these extracted line segments [6], uses SVM to classify these line segments. In [7,8], approaches to estimating the vanishing-point position are proposed and they use the road-tendency information provided by the vanishing point to estimate the optimal parameters of the lane model. A Conditional Random Function (CRF) model is also proposed to extract lane structure in [9].

### 2.2. Lane Detection with CNNs

Convolutional neural networks free us from designing handicraft features and rules, which have achieved state-of-art performance in many datasets. In [10], a multi-task network named VPG-net is proposed where multi-task training is proved that can improve the network performance. Fully convolutional networks for semantic segmentation are very suitable to solve lane-detection problems, and its encoder-decoder structure has been used in many research works, such as those of [11,12]. In [13], an instance-segmentation network is proposed, which can extract lane markings and divide them into different lane instances. In [14], a Spatial CNN (SCNN) is proposed, which can make the best of the relationship between pixels across rows and columns. Generative adversarial networks (GANs) are also studied in this field; for example, EL-GAN [15] uses GANs and embedding loss to train an end-to-end network.

## 3. Single Frame: Primary Probabilistic Hough Space via Lane Markings Extraction

In this section, a primary probabilistic Hough space is constructed by the line-segment extraction and classification. Firstly, a combination of Hough Transform and Random Sample Consensus (RANSAC) paradigm algorithm is employed to extract line segments efficiently. Then, the proposed CNN is used to classify these line segments and construct the primary probabilistic Hough space by using the output confidence of each line segment.

### 3.1. Line Segments Extraction by Hough Transform and RANSAC

Traditional Hough Transform actually leads to extensive computation cost because of its large voting range of direction which usually ranges from 0 to 360 degrees. An efficient Hough Transform [1] is used in this paper via the employment of edge direction to limit the voting range of direction. Defining the edge direction as ϕ and setting *H(ρ,θ)* as the Hough space, *c* represents the column number in image and *r* represents the row number, θ is limited by the right part of Equation (1). δ is set to 1 (degree) in this paper. This approach can make the extraction of line segments more efficient.

(1) ρ=c∗cos(θ)+r∗sin(θ)θ∈[ϕ−δϕ+δ]

**Figure 2:**
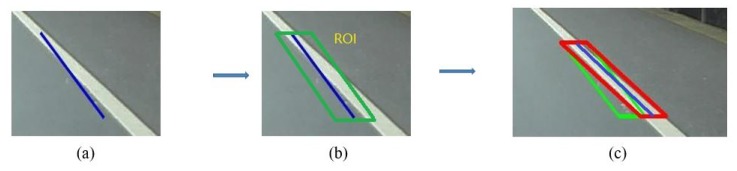
(**a**) A line segment disturbed by edge noise. (**b**) The original ROI which is proposed by the line segment. (**c**) The result of RANSAC. (green ROI: provided by the line segment before revision; red ROI: provided by the line segment after revision).

However, these line segments extracted by Hough Transform are easily influenced by noisy edge map as shown in Figure 2. A revision process is carried out by RANSAC. These line segments provide RANSAC with numbers of Regions of Interest (ROI) which are extracted from the neighbor of themselves, and RANSAC is then used to extract better line segments in these regions. Detailed information is described by Algorithm 1.

**Algorithm 1** Revising line segments by RANSAC: *R* represents ROI. (P1,P2) are two edge points randomly extracted from R. Defining *l* is the original line segment. *k* represents the slope of *l* and *b* is the bias, *n* is the number of iterations(n=40), lf is the final output**Input:***R,l:(k,b)* **Output:***lf*    **function**REVISE(R,l)          **while***n***do**               (P1,P2)←GettwoedgepointsrandomlyfromR               $\hat{l}:\left(\hat{k},\hat{b}\right)\leftarrow U\;se\;\left(P1,P2\right)\;to\;fit\;straight\;line$               **if**$\hat{l}\;has\;less\;outliers\;than\;l$**then***l* = $\hat{l}$:($\hat{k},\hat{b}$)               **end if**               n=n−1          **end while**          *lf* = *l*    **end function**

### 3.2. Constructing Primary Probabilistic Hough Space by Classification Networks

**Figure 3:**
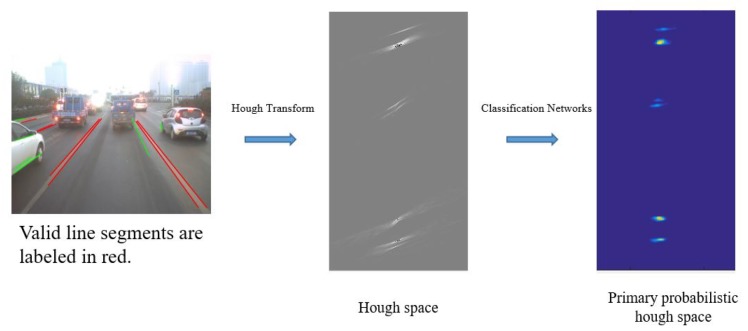
Primary probabilistic Hough space.

**Figure 4:**
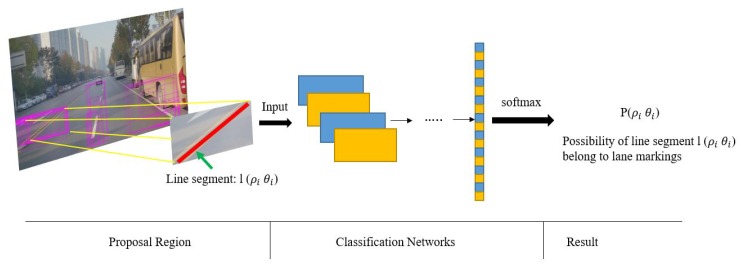
Process of line-segment classification by using the proposed network: the inputs are proposed by line segments and this classification network is used to measure each line segment by the metrics of possibility. The probabilistic Hough space is employed to record the confidence probability of each line segment.

After extraction of line segments, a post process is necessary to eliminate false detections such as those line segments overlapping fences. To solve this, a CNN-based classification network is proposed to classify line segments, and a probabilistic Hough space is constructed to record the confidence probability of each line segment. Valid line segments extracted from lane markings are labeled with high probability value in this proposed space (Figure 3). Table 1 shows the structure of the networks. The probabilistic Hough space is constructed by the outputs of the classification networks as demonstrated in Figure 4. A threshold ξ (which is set to 0.7) is used to choose the final valid line segments from the probabilistic Hough space.

Why do we need to construct the primary probabilistic Hough space? Indeed, we can choose the valid line segments by the proposed classification networks without constructing this Hough space. However, it is necessary to record the classification results of each frame when integrating sequential information to improve the performance of detection. Hough space is a convenient storage medium of storing the results of each frame.

**Figure 5:**
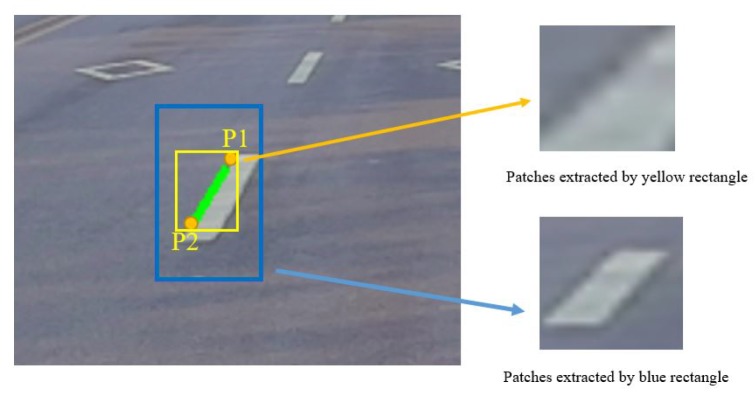
Yellow rectangle is proposed by the two endpoints (P1,P2) of line segments. Blue rectangle is proposed by two new calculated diagonal points by Equation (3).

The input of this network is provided by each line segment. The diagonal points of these input images will be calculated according to Equations (2) and (3). Firstly, $\left(x_{1},y_{1}\right)$ and $\left(x_{2},y_{2}\right)$ are two endpoints of line segment *l* in vehicle coordinate, and *k* is the slope of *l*. *W* is the max width of traffic lane markings. Two new endpoints $\left({\hat{x}}_{1},{\hat{y}}_{1}\right)$ and $\left({\hat{x}}_{2},{\hat{y}}_{2}\right)$ can be obtained according to Equation (3). Finally, these two new diagonal points can be projected into the image plane by Equation (2) and provide us with a reasonable patch as the blue one in Figure 5. In Equation (2), (x, y, z) is a point in the vehicle coordinates and ***H*** represents the perspective transformation matrix.

(2) $$s*\left(\begin{array}{c} c\\ r\\ 1 \end{array}\right)=H*\left(\begin{array}{c} x\\ y\\ z\\ 1 \end{array}\right)$$

(3) $$\begin{array}{c} \left({\hat{x}}_{1},{\hat{y}}_{1}\right)=\left(x_{1},y_{1}\right)+\left(-\frac{k}{\left|k\right|}\times w,-\frac{w}{\left|k\right|}\right)\\ \left({\hat{x}}_{2},{\hat{y}}_{2}\right)=\left(x_{2},y_{2}\right)+\left(\frac{k}{\left|k\right|}\times w,\frac{w}{\left|k\right|}\right) \end{array}$$

**Figure 6:**
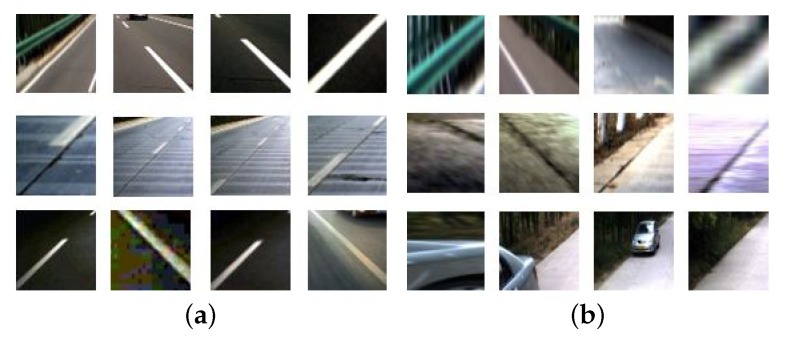
(**a**) Positive samples. (**b**) Negative samples.

A dataset was established for training and testing as illustrated in Figure 6. The positive samples are line segments which truly belong to traffic lane markings and negative samples are false line segments. A total of 50,000 pictures were collected.

## 4. Sequential Frames: Filtered Probabilistic Hough Space via IMU and Vision Data

**Figure 7:**
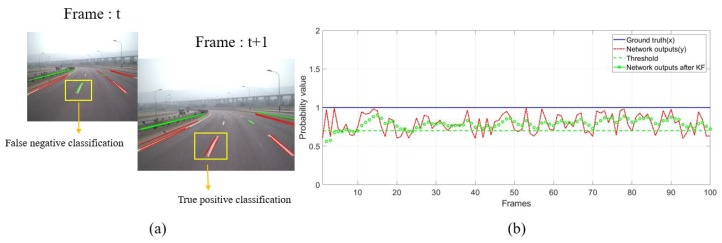
(**a**) due to the vehicle movement and the classification error of the networks, the same line segment has different classification results at time *t* and *t* + 1. (**b**) plotting the probability values before and after Kalman filtering.

The existence of lane markings is consistent in the sense that they rarely abruptly appear or disappear in the view. Therefore, it is very likely for a line segment with a sudden appearance or disappearance to be false. On the contrary, if valid line segments appear in the same place often, the corresponding positions will keep high probability values for line segments. However, the primary probabilistic Hough space mentioned above is easily disturbed by occlusion, movement of vehicle and classification error (Figure 7). Thus, a Kalman Filter is used to smooth the primary probabilistic Hough space across sequence frames in this section. Movement information provided by IMU is applied to make the line segments extracted at different times aligned in the current Hough space.

### 4.1. Filtering Primary Hough Space with Kalman Filter

Kalman filtering makes the filtering process more efficient by using the Markov Assumption. Setting *x* as the probability value of a line segment *l* and *y* as the output confidence of the classification networks. Theoretically, *x* is equal to 1 if *l* is valid, otherwise *x* is equal to 0. The state-transition matrix *A* and the observation matrix *C* are both set to be a unit matrix. The noise matrix *B* is a zero matrix as the attribute of the *l* should be kept consistent with the previous frames. *D* is the observation noise caused by vehicle movement and classification error of networks. Equation (4) are the state equation for Kalman filtering.

(4) $$\begin{array}{c} x_{t}=A*x_{t-1}+B\\ y_{t}=C*x_{t}+D \end{array}$$

The filtered probabilistic Hough space describes the probability of whether a line segment belongs to traffic lane markings or not and is more reliable than the primary probabilistic Hough space.

### 4.2. Aligning Previous Line Segments in the Current Hough Space

**Figure 8:**
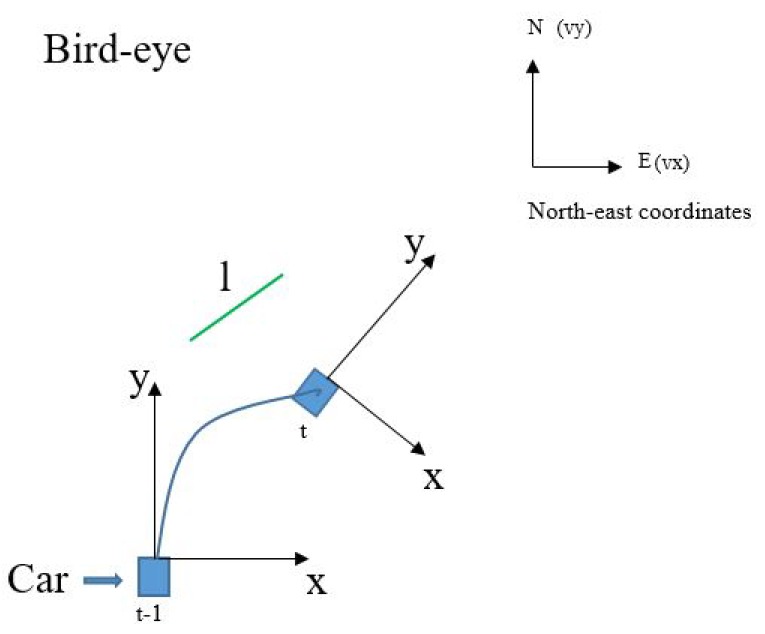
The line segment *l* has different positions in vehicle coordinate and Hough space at different times. Velocity *V* and acceleration *A* are measured in north-east coordinates.

As shown in Figure 8, line segment *l* has different positions at different times because of the movement of the vehicle. Therefore, it is necessary for Kalman filtering to obtain its observed value *y* from sets of probabilistic Hough spaces which extracted at different times, meaning alignment of $l_{t-1}\left(\rho_{t-1},\theta_{t-1}\right)$ and $l_{t}\left(\rho_{t},\theta_{t}\right)$ should be performed in the current Hough space.

To begin with, $l_{t-1}\left(\rho_{t-1},\theta_{t-1}\right)$ from previous vehicle coordinate needs to be projected into the current coordinate based on IMU information including velocity *V*=*(vx, vy, vz)*, acceleration *A*=*(ax, ay, az)* and Euler Angle *(α, β, γ)*. Rotation matrix and transition matrix are calculated by Equations (5) and (6), respectively. Defining ($\left[x_{t-1}^{1},y_{t-1}^{1},z_{t-1}^{1}\right]$,$\left[x_{t-1}^{2},y_{t-1}^{2},z_{t-1}^{2}\right]$) as the two endpoints of *l* at time t−1 in the vehicle coordinates. Its position at time *t* can be calculated by Equation (7) (*i* = 1, 2). Finally, $\left(\rho_{t},\theta_{t}\right)$ is solved by perspective mapping (Equation (2)) and Hough Transform (Equation (8)).

(5) $$\begin{array}{c} R\left(\alpha ,\beta ,\gamma \right)=\left(\begin{array}{ccc} 1 & 0 & 0\\ 0 & \cos\alpha & \sin\alpha \\ 0 & -\sin\alpha & \cos\alpha \end{array}\right)*\left(\begin{array}{ccc} \cos\beta & 0 & -\sin\beta \\ 0 & 1 & 0\\ \sin\beta & 0 & \cos\beta \end{array}\right)\\ {}*\left(\begin{array}{ccc} \cos\gamma & \sin\gamma & 0\\ -\sin\gamma & \cos\gamma & 0\\ 0 & 0 & 1 \end{array}\right) \end{array}$$

(6) $$\Delta T=\int_{t-1}^{t}V\left(t\right)+\frac{1}{2}\times A(t)\times t^{2}dt$$

(7) $$\left[\begin{array}{c} x_{t}^{i}\\ y_{t}^{i}\\ z_{t}^{i} \end{array}\right]=R\left(\Delta \alpha ,\Delta \beta ,\Delta \gamma \right)*\left[\begin{array}{c} x_{t-1}^{i}\\ y_{t-1}^{i}\\ z_{t-1}^{i} \end{array}\right]+R\left(\alpha_{t},\beta_{t},\gamma_{t}\right)*\Delta T$$

(8) $$\begin{array}{c} \theta_{t}=\arctan\left(-\frac{r^{1}-r^{2}}{c^{1}-c^{2}}\right)+\frac{\pi }{2}\\ \rho_{t}=c^{1}*\cos\left(\theta \right)+r^{1}*\sin\left(\theta \right) \end{array}$$

**Figure 9:**
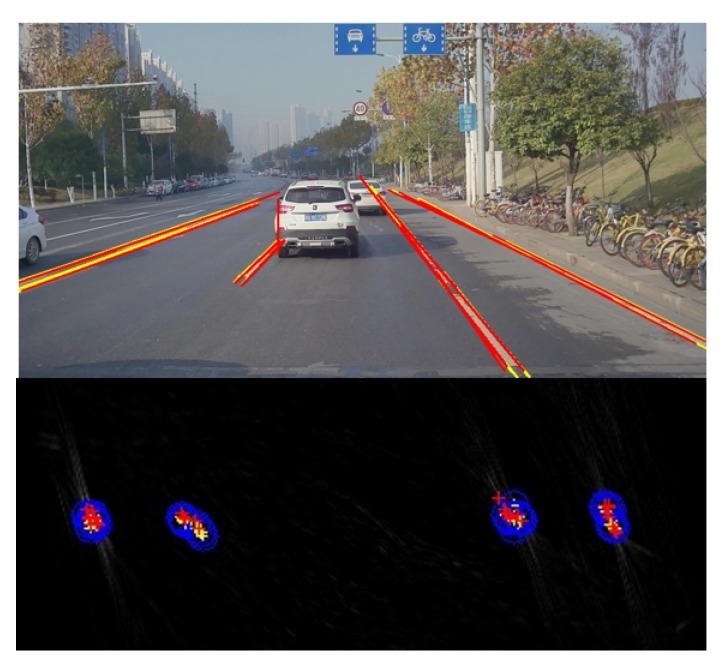
The result of alignment during neighbor frames. The current detections are labeled in red and the previous results (after alignment) are labeled in yellow. The bottom part shows the result in the Hough space and the blue circles represent the range of alignment error.

Despite the effort described above, precision alignment is hard to achieve due to some factors such as the noise of IMU. So we regard all the {*($\hat{\theta },\;\hat{\rho }$)*} (calculated by Equation (9)) as the alignment results of $l_{t-1}\left(\rho_{t-1},\theta_{t-1}\right)$ at time *t*. The alignment error *r* is set to be 49 in this paper. The final result is demonstrated by Figure 9. The current detections are labeled in red and the previous results (after alignment) are labeled in yellow.

(9) $${\left(\hat{\theta }-\theta_{t}\right)}^{2}+{\left(\hat{\rho }-\rho_{t}\right)}^{2}<=r$$

### 4.3. Final Lane Fitting Using the Result of Sequential Frames

**Figure 10:**
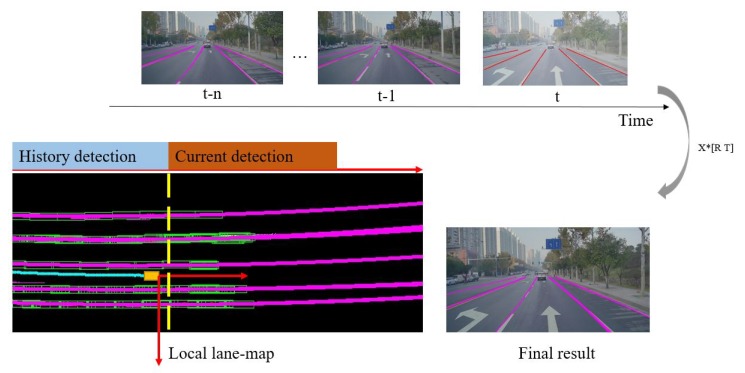
Local lane-map is constructed by connecting those recorded results from *t-n* to *t* in the same vehicle coordinate. It makes the final output more stable by providing useful information for the fitting stage in a larger spatial and time scale than single frame.

By connecting valid line segments detected across frames as illustrated in Figure 10, the problem of lane fitting can be solved with extensive sequential information. To give the final outputs, a region-growth algorithm is used to divide these foreground points into different lane instances and a parabolic model is used to fit each lane in the current vehicle coordinate. Figure 10 shows the full process mentioned above. To limit the risk of over-fitting, L2 norm is added into the loss function as Equation (10) where $\alpha_{1}$ (set to be 0.9) and $\alpha_{2}$ (set to be 0.3) are tradeoff coefficients.

(10) $$E=\alpha_{1}\sum {\left(ax^{2}+bx+c-y\right)}^{2}+\alpha_{2}{∥a∥}^{2}$$

## 5. Results and Discussion

**Figure 11:**
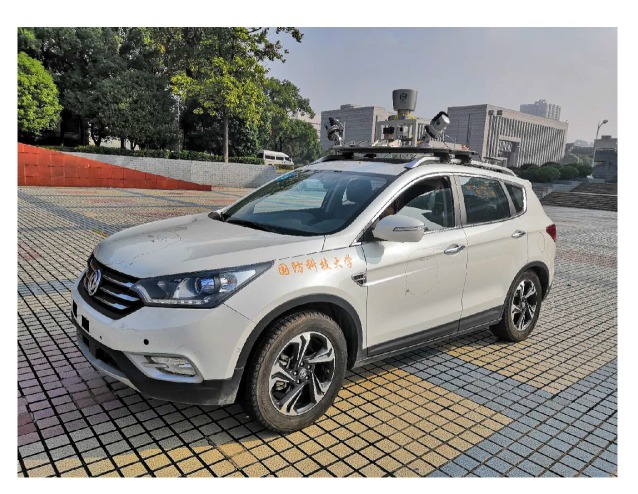
The experimental vehicle produced by the Dongfeng Motor Corporation.

We run the proposed algorithm on an Intel(R) Core(TM) i7-7700HQ 2.80GHz CPU with a NVIDIA GTX1050ti GPU. The average total time cost is 52.3 ms. Processing steps mentioned in Section 3 cost 22 ms (Hough Transform: 10.6 ms, classification networks (one-line segment): 0.3 ms). Processing steps mentioned in Section 4 cost 25 ms (Kalman filtering:2ms, Alignment: 9 ms, lane fitting: 11 ms). One camera and one IMU are employed. The type of the camera is OV10650 and the IMU is Epson G320. Figure 11 shows the vehicle used to carry out the experiments.

This section is divided into two parts. In the first part, detailed analysis of the performance of the classification networks will be introduced. Experiments about the filtered probabilistic Hough space will be discussed in the second part, where the fusion of IMU and vision is employed.

### 5.1. Performance of the Classification Networks

The performance of the classification networks was tested under Caltech dataset [16]. This dataset contains four video sequences all sampled in urban areas. Easy conditions and challenging scenarios are all included, such as shadows or writing. Please note that only two lines in the current lane were detected in this part. Comparison between the used algorithm and other ones was carried out using this dataset based on the metrics of Accuracy Rate (AR) and False Negative Rate (FNR).

**Figure 12:**
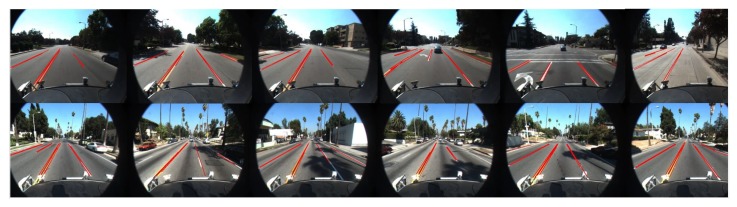
Performance with Caltech datasets.

Figure 12 demonstrates the test result of the proposed method with the Caltech dataset. Table 2 shows that the proposed method for line segments extraction and classification can achieve a more satisfying performance compared to Niu’s method.

### 5.2. Performance of the Filtered Probabilistic Hough Space

To employ sequence information for lane detection, the information provided by vision and IMU needs to be integrated. More specifically, Euler angle and velocity obtained from IMU were used to align history results in the same coordinate. This alignment helps to match the same line segments at different times, which is necessary for Kalman filtering at a later stage. The filtered probabilistic Hough space has a higher reliability compared to the primary probabilistic Hough space.

**Figure 13:**
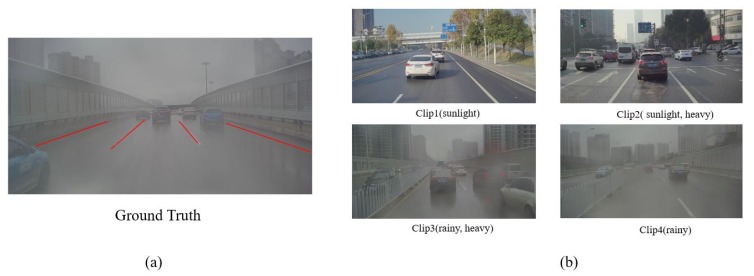
(**a**) Ground truth is labeled in the form of line segments. (**b**) Four parts of data are chosen to test the algorithm: clip1 (sunlight), clip2 (sunlight, heavy), clip3 (rainy, heavy), clip4 (rainy).

To evaluate our algorithm, four parts of the road data (Figure 13b) were chosen to test the performance of our method with the measurement metric of accuracy(ACC). Those annotated pictures are labeled in the form of line segments as shown in Figure 13a.

A threshold ξ( set to be 0.7) was used to choose the final valid line segments from the filtered probabilistic Hough space (Equation (11)).

(11) $$Attribute=\left\{\begin{array}{cc} valid, & p(\rho ,\theta )\geq \xi \\ false, & p(\rho ,\theta )<\xi . \end{array}\right.$$

Table 3 lists the accuracy of classification when using the primary probabilistic Hough space and the filtered probabilistic Hough space. It is proven that the filtering process can evidently enhance the accuracy of line-segment classification.

**Figure 14:**
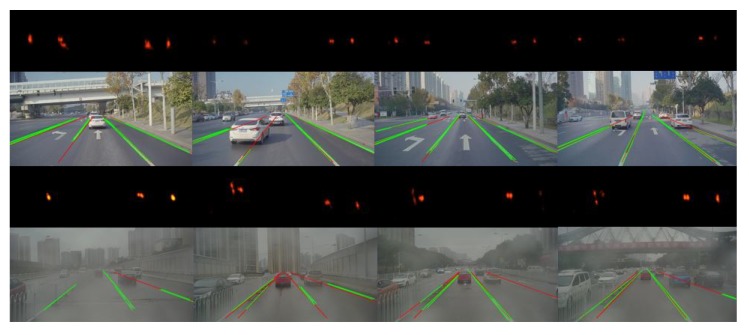
The first and third rows show the probabilistic Hough space where the points with high brightness represent the possible valid line segments. The second and fourth rows show the corresponding result of line segments extraction where green line segments are the result of detection and red ones are the result of tracking.

Figure 14 illustrates the result of line-segment detection and tracking. The first and third rows in this figure are the corresponding probabilistic Hough space where the points with high brightness represent the valid line segments.

Table 4 is the comparison of the performance between the proposed approach in this paper and Neven’s method [13], demonstrating that, most of the time, our method outperforms Neven’s, especially in terms of false-positive rate due to the use of sequential information.

**Figure 15:**
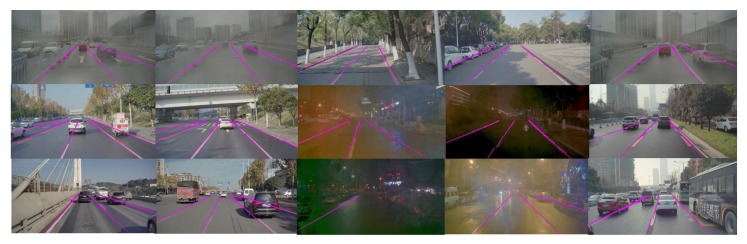
Detection under different scenarios.

**Figure 16:**
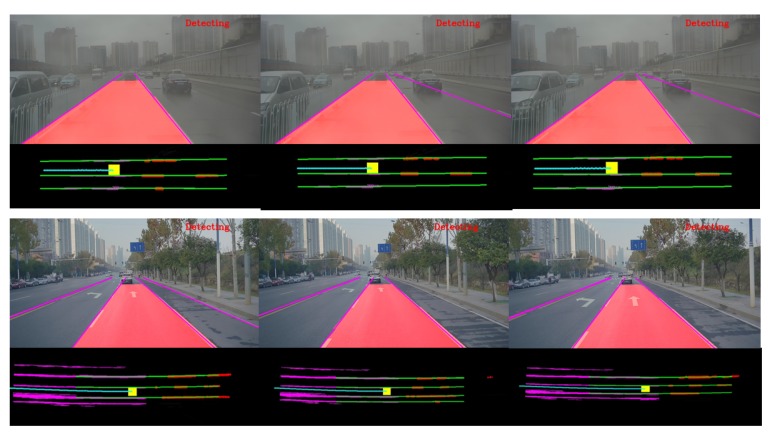
The final results are displayed in the image coordinates and vehicle coordinates. In the second and fourth rows, the yellow rectangle represents the center of the vehicle. Line segments detected in the past are labeled in purple and those extracted from the current frame are labeled in red. The results of lane fitting are labeled in green. The cyan points represent the trace of the vehicle which are calculated by the IMU data.

By connecting the line segments stored in the past, the problem of lane fitting could be solved with more history information. The results of the proposed approach are showed by Figure 15. Figure 16 describes the final results in the image coordinates and vehicle coordinates which would make it more intuitive to understand the proposed approach.

## 6. Conclusions

In this paper, a multi-stage Hough space calculation was proposed for a lane-detection task by the fusion of vision and IMU. An efficient Hough Transform and a classification CNNs were introduced to extract and classify line segments from images. By using the outputs of the proposed classification networks, a novel primary probabilistic Hough space was constructed. Kalman filtering was later employed to smooth the probabilistic Hough space across frames for the purpose of eliminating the disturbance from occlusion, movement of vehicle, and classification error. After that movement, information provided by the IMU was applied for aligning the previously detected line segments with the current ones in the current Hough space. The filtered probabilistic Hough space was finally used to clean out line segments with low probability values (threshold was set as 0.7) which were considered false, and to output those with high probability values as the final valid line segments. Though the current method already has a better performance compared to various existing ones mentioned in the paper, more developments are still being sought to further improve the algorithm in the future.

## Figures and Tables

**Table 1:** Structure of our classification network.

Layer Index	1	2	3	4	5	6
Layer Name	Data	Conv+Relu	Pooling	Conv+Relu	Interp	Pooling
Output Size	(64,64,3)	(62,62,40)	(31,31,40)	(29,29,20)	(28,28,20)	(14,14,20)
Layer Index	7	8	9	10	11	12
Layer Name	Conv	Pooling	Conv	Inner-Product	Inner-Product	Softmax
Output Size	(10,10,20)	(5,5,20)	(1,1,50)	(1,1,500)	(1,1,2)	(1,1,2)

**Table 2:** Performance of Different Algorithms with Caltech Dataset.

Clip	Total	Niu’s Method [4]			Our Method	
		AR(%)	FN(%)		AR(%)	FN(%)
cordova1	466	92.2	5.4		97.25	2.7
cordova2	472	97.7	1.8		97.05	1.2
washington1	639	96.9	2.5		95.84	3.7
washington2	452	98.5	1.7		95.63	3.1

**Table 3:** Accuracy of the line segments extraction.

Datasets	clip1	clip2	clip3	clip4
Filtered probabilistic Hough space (sequential frames)	0.95	0.93	0.91	0.94
CNNs-based classification (single frame)	0.91	0.89	0.88	0.92

**Table 4:** Performance of each algorithm under our own dataset.

Clip	Total	Neven’s Method [13]			Our Method	
		TP(%)	FP(%)		TP(%)	FP(%)
part1	927	61.8	6.7		72.2	0.6
part2	174	78.2	38.5		72.9	1.5
part3	647	83.6	6.1		87.3	1.7
part4	713	82.5	5.9		76.5	0.1
